# Supplementary material for: Trends in the Consumption of Antidepressant Drugs before and during the COVID-19 Pandemic in the Canary Islands, Spain: The Case of the Province of Las Palmas
Source: Healthcare (Basel). 2023 May 15;11(10):1425. doi: 10.3390/healthcare11101425 (PMC10218601; doi:10.3390/healthcare11101425)
Supplement: Supplementary file 1 [file healthcare-11-01425-s001.zip › healthcare-2388258-supplementary.pdf]

Table S1 (A)

ANOVA results on total DID variation data expressed per quarter and year according to data in Figure 2 for all islands.

Source of variation	Df	Sum of Square	Mean Square	F-value	Pr(>F)
Quarter	1	306	306.0	54.828	1.0e-09 *
Island	2	35334	17666.8	3165.57	<2.2e-16*
Quarter x Island	2	257	128.4	23.01	6.42e-08*
Residuals	53	296	5.3		

Coefficient:	Estimate	Pr(>|t|)	2.5%	97.5%
Intercept [1]	28.74	$<2.0 \times 10^{-6}$ *	26.54	30.94
Quarter [2]	0.320	9.53×10^{-4} *	0.137	0.504
Island_GC	45.65	$<2.0 \times 10^{-6}$ *	42.49	48.81
Island_LZ	2.895	**0.0677**	-0.218	6.00
Quarter x Island_GC	0.819	$<2.0 \times 10^{-6}$ *	0.548	1.089
Quarter x island_LZ	0.034	**0.794**	-0.226	0.294

Residual standard error: 2.362 on 53 degrees of freedom; adjusted R-squared: 0.9911

[1]: Reference value used by the R-program. In this case, the level of dispensation for the island of Fuerteventura

[2]: Total DID variation rate per quarter and year

(GC = Gran Canaria; FV = Fuerteventura; LZ = Lanzarote)

Table S1 (B)

ANOVA results for the islands of Gran Canaria, Fuerteventura and Lanzarote for 2016-2020 according to the annual consumption data (expressed as total DID per year) shown in Table 2.

Source of variation	Df	Sum of Square	Mean Square	F-value	Pr(>F)
Year	1	195.0	195.0	367.2	$<2.0 \times 10^{-6}$ *
Island	2	9258.8	4629.4	8719.6	$<2.2 \times 10^{-6}$ *
Year x Island	2	82.7	41.3	77.848	$<2.2 \times 10^{-6}$ *
Residuals	9	4.8	0.5		

Coefficient:	Estimate	Pr(>|t|)	2.5%	97.5%
Intercept [1]	28.27	$<2.0 \times 10^{-6}$ *	26.54	30.94
Year [3]	1.277	3.6×10^{-4} *	0.756	1.798
Island_GC	43.40	$<2.0 \times 10^{-6}$ *	40.96	45.85
Island_LZ	2.652	0.0365 *	0.207	5.097
Year x Island_GC	3.617	1.49×10^{-6} *	2.88	4.35
Year x island_LZ	0.200	**0.614**	-0.089	0.798

[3]: Total DID variation rate per year
